# Contrast agents in dynamic contrast-enhanced magnetic resonance imaging

**DOI:** 10.18632/oncotarget.16482

**Published:** 2017-03-22

**Authors:** Yuling Yan, Xilin Sun, Baozhong Shen

**Affiliations:** ^1^ Molecular Imaging Research Center (MIRC), Harbin Medical University, Harbin, Heilongjiang, China; ^2^ TOF-PET/CT/MR Center, The Fourth Hospital of Harbin Medical University, Harbin, Heilongjiang, China; ^3^ Molecular Imaging Program at Stanford (MIPS), Department of Radiology, Stanford University School of Medicine, Stanford, California, USA

**Keywords:** DCE-MRI, contrast agent, macromolecular contrast agent, nanoparticle, low molecular contrast agent

## Abstract

Dynamic contrast-enhanced magnetic resonance imaging (DCE-MRI) is a noninvasive method to assess angiogenesis, which is widely used in clinical applications including diagnosis, monitoring therapy response and prognosis estimation in cancer patients. Contrast agents play a crucial role in DCE-MRI and should be carefully selected in order to improve accuracy in DCE-MRI examination. Over the past decades, there was much progress in the development of optimal contrast agents in DCE-MRI. In this review, we describe the recent research advances in this field and discuss properties of contrast agents, as well as their advantages and disadvantages. Finally, we discuss the research perspectives for improving this promising imaging method.

## INTRODUCTION

**Figure 1:**
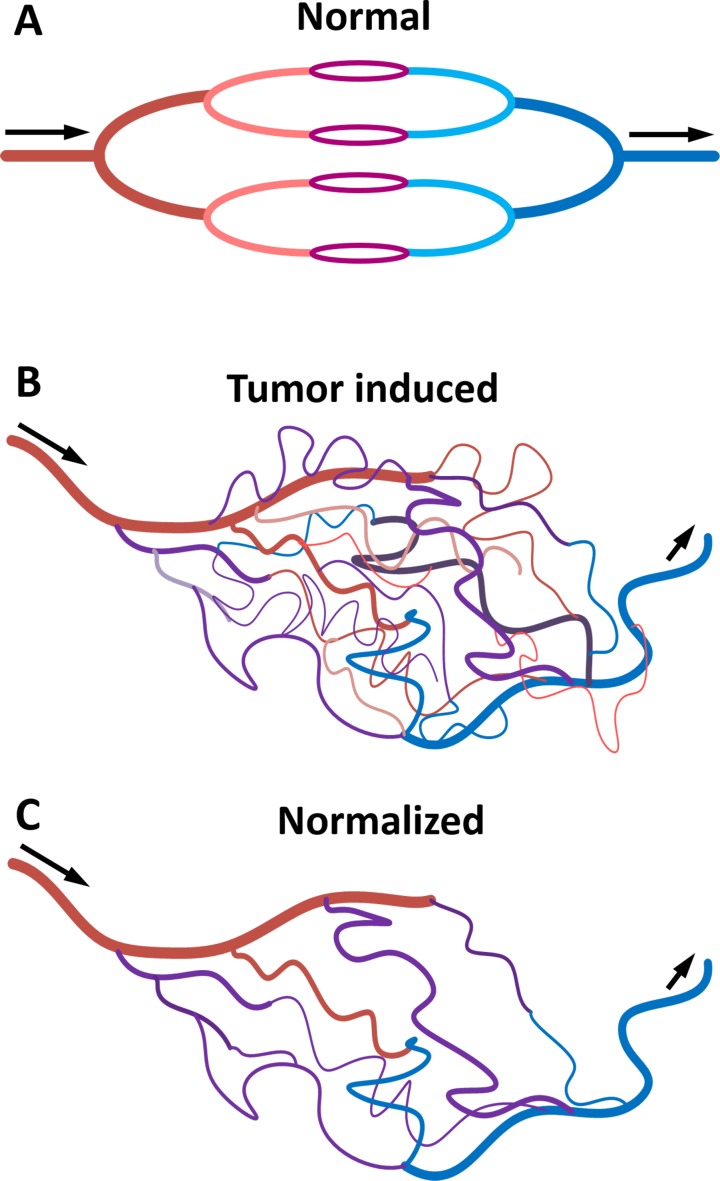
Diagram representing blood vessels in normal tissue A., tumor tissue B. and tumor tissue after treatment with anti-angiogenic drugs C Tumor vessels have active angiogenesis, high permeability, hypoxia and the blood flow is chaotic and slow. After anti-angiogenic therapy, tumor blood vessels recover their normal functions.

Cancer is one of the leading causes of death worldwide. The improvement of diagnosis, treatment and prognosis estimation are central in cancer research. In recent years, traditional cancer treatments such as surgery, chemotherapy and radiotherapy have increasingly been replaced or complemented with targeted therapy. It is now well established that the physiology and morphology of malignant tumors are different from those of normal tissue and benign lesions. In particular, several studies have demonstrated that malignant tumors have abnormal vessels with increased permeability and density, as well as various defects at the morphological and molecular level [1–3] (Figure 1). Tumor vessels are essential for cancer growth and metastasis because they provide nutrients and remove metabolic waste from cancer cells. It is therefore possible to use blood vessel morphology and properties to diagnose cancer and monitor therapy response. Indeed, therapies targeting vascularization are extremely popular in cancer treatment and it has even been suggested they represent a potential method for curing cancer [4–6].

Bevacizumab is a monoclonal antibody that specifically recognizes and binds to vascular endothelial growth factor (VEGF), thus preventing it from activating the VEGF receptor. Sunitinib is small molecule that binds VEGF receptors (VEGFRs) on the surface of endothelial cells to block VEGFR tyrosine kinase activity and blocks VEGFR1-3, PDGFR, KIT and FLT3 signal. Sorafenib inhibits signal of VEGFR-2, VEGFR-3, RAF, PDGFR, KIT and RET, These anti-angiogenic drugs are currently used to treat a variety of cancers with considerable success [7, 8]. However, needle biopsies, morphological imaging and biochemical indicators are typically needed for performing diagnostics based on vascularization, and for monitoring therapeutic effects and estimating prognosis of cancer patients before, during and after vascular targeting therapy. As these methods are invasive and have low sensitivity and specificity, developing a specific and effective method to monitor vascular change during anti-angiogenesis therapy is fundamental for improving cancer treatment.

Dynamic contrast-enhanced magnetic resonance imaging (DCE-MRI) is a noninvasive method for assessing vascular physiological characteristics. Contrast agents play a key role in this imaging technique. There are several types of contrast agents currently available, but the most commonly used in the clinic are Gd (III)-based contrast probes. At least nine formulations of Gd-containing contrast agents have been approved for human use in the United States, and they are assisting more than 10 million MRI scans every year [9]. Moreover, a variety of contrast agents, each with their own distinctive properties for DCE-MRI, are also being widely used in preclinical studies. It has been demonstrated that the choice of contrast agent influences DCE-MRI results, in particular, the agent size, chemical property and pharmacokinetic may affect the reliability of this technique [10]. It is therefore essential to select reliable agents. However, while extensive research efforts have been invested into understanding the effects of different contrast agents on DCE-MRI, there is an ongoing debate as to whether low molecular weight agents are suited for DCE-MRI or macromolecular weight probes should be used instead and even nanomaterials have been explored in DCE-MRI. Low molecular agents are more commonly used, but they lead to an over-estimation of vessel permeability, macromolecular contrast agents are undergoing preclinical research. Thus, presently there is no consensus on which is the optimal agent for DCE-MRI and it remains unclear how to select the appropriate contrast agent for a particular application. This is the first review about contrast agents used in DCE-MRI. In this review, we introduce several types of contrast agent and discuss the recent research advances in the field, as well as the advantages and disadvantages of each agent and the perspectives for possible applications. Finally, we speculate on which may be the most appropriate contrast agent for DCE-MRI in the future.

## MATHEMATICAL MODEL

**Figure 2:**
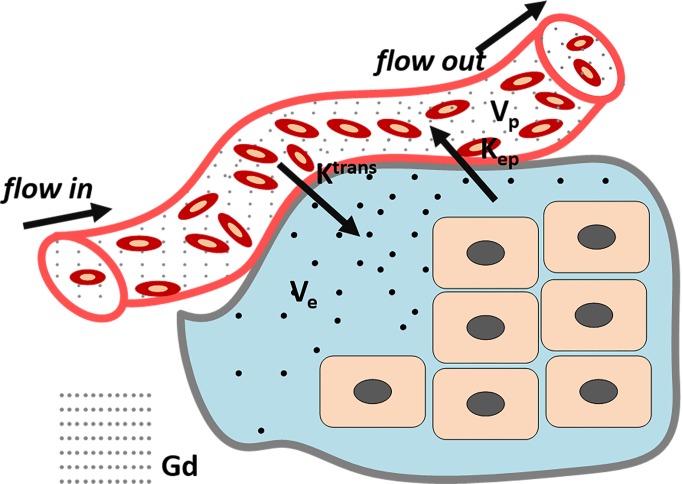
Tofts Model: classical DCE-MRI model (K^trans^: the transfer constant from vascular to extravascular extracellular space (EES); K_ep_: Rate constant between the EES and the blood plasma; V_e_: The fractional tissue EES (light green). V_p_: The fractional plasma volume (white)

DCE-MRI with appropriate pharmacokinetics mathematical model can evaluate angiogenesis precisely. With the knowledge of abnormal tumor microcirculation system, DCE-MRI can dynamically and continuously monitor distribution of agents in the lesion, and then through a post-processing system to acquire quantitative and semi-quantitative parameters to assess the tumor tissue vascular density, integrity and permeability. Contrast agents leak from the intravascular to the extravascular extracellular space (EES), resulting in a signal increase on T1-weighted MRI. As the rate of extravasation depends on vascular surface area, permeability and blood perfusion, and the enhancement of region-of-interest (ROI) can be recorded in a curve as a function of time, DCE-MRI offers a dynamic physiological picture of the tumor blood vessels. There are two types of methods for DCE-MRI data analysis. The first is a semi-quantitative method, in which different parameters characterizing the shape of the normalized signal intensity (SI) time curve can be extracted. Specifically, the (i) area under curve expresses the amount of ROI increase over a defined period of time (usually from starting increment of the SI-time curve to 60 or 90 s); the (ii) maximum of SI or peak wash-in slope determines the velocity of enhancement, and it is calculated as the maximum change in enhancement per unit time, usually from 20% to 80 %; the (iii) range of the increment curve enhancement ratio (SI maximum-SI base line/SI base line) of the enhancing curve and the (iv) mean transit time (MTT) represents the mean time for blood to perfuse a region of tissue, and is affected by the blood volume and blood flow in the region under analysis [11–13]. Although this semi-quantitative method is widely used because it does not require any models, the analysis results can be ambiguous or inaccurate as they may be affected by the inspection equipment, injection technique or contrast agent. Moreover, the descriptive parameters extracted with this method cannot offer any physiologic insight into the behavior of the tumor vessels. For these reasons, this method has gradually been replaced by quantitative approaches, which are based on modelling concentration changes of the contrast agent with pharmacokinetic methods. The first pharmacokinetic model was proposed in the early 1990s by Larsson, Tofts, Brix et, al and was named the ‘Tofts model’ after one of his authors (Figure 2). Since then several other models have been proposed. Up to now, the pharmacokinetic models used in DCE-MRI are reviewed by Steven P. Sourbron [14]. In classical pharmacokinetic model, SI is converted into concentration, and then concentration *vs* time curves are fitted using a bicompartmental model (vessels and EES). Finally, the following classical parameters can be extracted: (1) K^trans^ denotes the vascular-to-EES transfer constant and is affected by vascular permeability, blood perfusion and vascular surface area; (2) K_ep_ represents the EES-to-blood plasma rate constant; (3) V_e_ indicates interstitial space and (4) V_p_ denotes the volume of blood vessels [11, 12, 15, 16]. By selecting an appropriate pharmacokinetics model, it is possible to use DCE-MRI to evaluate vessel characteristics. Currently, DCE-MRI is widely used in assessing tumor angiogenesis [17–19] and therapy-induced microvascular changes [20, 21], as well as for estimating cancer prognosis *in vivo* [22, 23]. With appropriate pharmacokinetic model and proper contrast agent is crucial for DCE-MRI development.

## DCE-MRI WITH LOW MOLECULAR CONTRAST AGENTS

**Figure 3:**
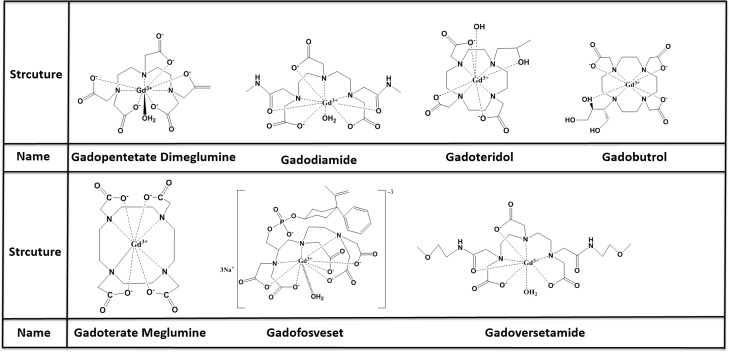
structure and name of low olecular MR contrast agents

MRI contrast agents can be classified by molecular weight into macromolecular or low molecular contrast agents, the latter typically measuring below 10 kD and being the most widely used. Seven low molecular contrast agents have been approved by the FDA (2013): Gadopentetate Dimeglumine, Gadoterate Meglumine Gadodiamide, Gadoteridol, Gadobutrol, Gadoversetamide and Gadofosveset are representative of these agents and are commonly used in the clinic for the diagnosis, monitoring and prognosis estimation of many types of cancer including breast cancer, brain glioma, lung cancer, hepatocellular carcinoma, kidney cancer, prostatic cancer, ovarian carcinoma and so on [4, 6, 10, 17, 18, 21, 22, 24–29](Figure 3). Gadopentetate dimeglumine (Gd-DTPA) as the representative of low molecular contrast agents, shows great potential in tumor management with DCE-MRI. some new low molecular contrast agents are currently being tested for DCE-MRI in preclinical. This review will discuss these agents’ recent developments.

### DCE-MRI with Gd-DTPA in tumor diagnosis

Malignant tumors have distinctive microvessels with different physiology from those of normal lesions. DCE-MRI can reveal the tumour vessels characteristics noninvasively and thereby distinguish malignant from benign lesions. For instance, it was shown that DCE-MRI with Gd-DTPA could identify asymptomatic or symptomatic myeloma, specifically, it detected increased microcirculation parameters in multiple myeloma and monoclonal gammopathy of unknown significance patients [30]. Moreover, DCE-MRI with diffusion weight imaging (DWI) showed promise in differentiating myeloproliferative disorder from benign lesions with high accuracy and sensitivity [31], DCE-MRI with Gd-DTPA evaluate oral squamous cell carcinoma stage, advanced stage with lower K^trans^ value [32]. There is another report about DCE-MRI with Gd-DTPA can distinguish mucosa-associated lymphoepithelial lesion from benign lymphoepithelial lesion, semi-quantitative parameters time to peak (TTP) and time to start in the two lesions are significant different [33]. Jia et al explored the potential of three-dimensional contrast enhanced ultrasound (3D-CEUS) and DCE-MRI for evaluating breast tumor angiogenesis by correlating their diagnostic capabilities with biological factors, and found that both 3D-CEUS and DCE-MRI with Gd-DTPA are suitable for detecting tumor angiogenesis [34]. In a retrospective study on solid pancreatic lesions, it was further demonstrated that DCE-MRI with Gd-DTPA is a promising method for detecting particular characteristics of pancreatic diseases [35]. Moreover, DCE-MRI with other imaging methods could evaluate angiogenesis in a nude rat model of breast cancer bone metastasis [28].

### DCE-MRI with Gd-DTPA in treatment monitoring

Traditional methods for treating cancer include surgery, radiotherapy and chemotherapy, however, targeted therapies have been increasingly used with considerable success, in particular angiogenesis-targeted therapy. Contrary to traditional examination techniques such as biopsies, which cannot provide information regarding the effectiveness of angiogenesis-targeted therapy, dynamic contrast agents can monitor vascular changes continuously. Liu et al first used DCE-MRI with Gd-DTPA to monitor kinetic changes of therapeutic molecules in the brain resulting from focused ultrasound, and found that the kinetic increments detected by DCE-MRI correlated well with concentration changes in Evans Blue (EB)-albumin (coefficient of 0.74-0.89) [36]. This study suggested that DCE-MRI is a promising method for monitoring pharmacokinetics and pharmacodynamics *in vivo*.

Several preclinical studies demonstrated that DCE-MRI with Gd-DTPA is a method with great potential for assessing the effects of anti-angiogenesis drugs [4, 21]. Indeed, this method has been used for detecting pharmacokinetics and monitoring the effectiveness of anti-angiogenesis therapy in the treatment of cancer [37–40]. DCE-MRI with Gd-DTPA combined with serological angiogenic markers can predict the progression of residual ovarian cancer after cytotoxic therapy. Moreover, this method also detects early therapeutic effects, which could be valuable for distinguishing sensitive from non-sensitive patients and thus select those patients who may benefit from the treatment [25, 41].

**Figure 4:**
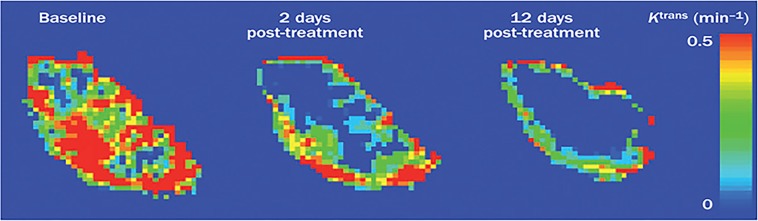
Example of three K ^trans^ parameter maps. The three K^trans^ maps showed are from a patient with a colorectal liver metastasis before and after treatment with bevacizumab. The distribution of K^trans^ is spatially heterogeneous with high values in the periphery and low values in the core. K^trans^ was reduced after 2 days and further reduced after 12 days of treatment when compared with baseline. Abbreviations: DCE-MRI, dynamic contrast-enhanced MRI; K^trans^, volume transfer constant between plasma and the extracellular extravascular leakage space

Additionally, over 100 preclinical trials have used DCE-MRI to monitor the effects of anti-angiogenic drugs by assessing the structure and function of the tumor vessels, thus highlighting the important role of DCE-MRI for drug development. Together these studies show that DCE-MRI with Gd-DTPA is a promising method to monitor tumor treatment [42] (Figure 4). However, care should be taken when using this method, for instance, in the application of multi parameters and quantitative parameters calculations, changes of multi-parameters DCE-MRI are necessary in treatment monitoring but not sufficient, most researches in clinical are single center data, small sample, especially using DCE-MRI multi parameters predicting survival of patients. So there is still need for more experiments to verify the important role of DCE-MRI in cancer patients monitoring

### DCE-MRI with Gd-DTPA in evaluating tumour prognosis

**Figure 5:**
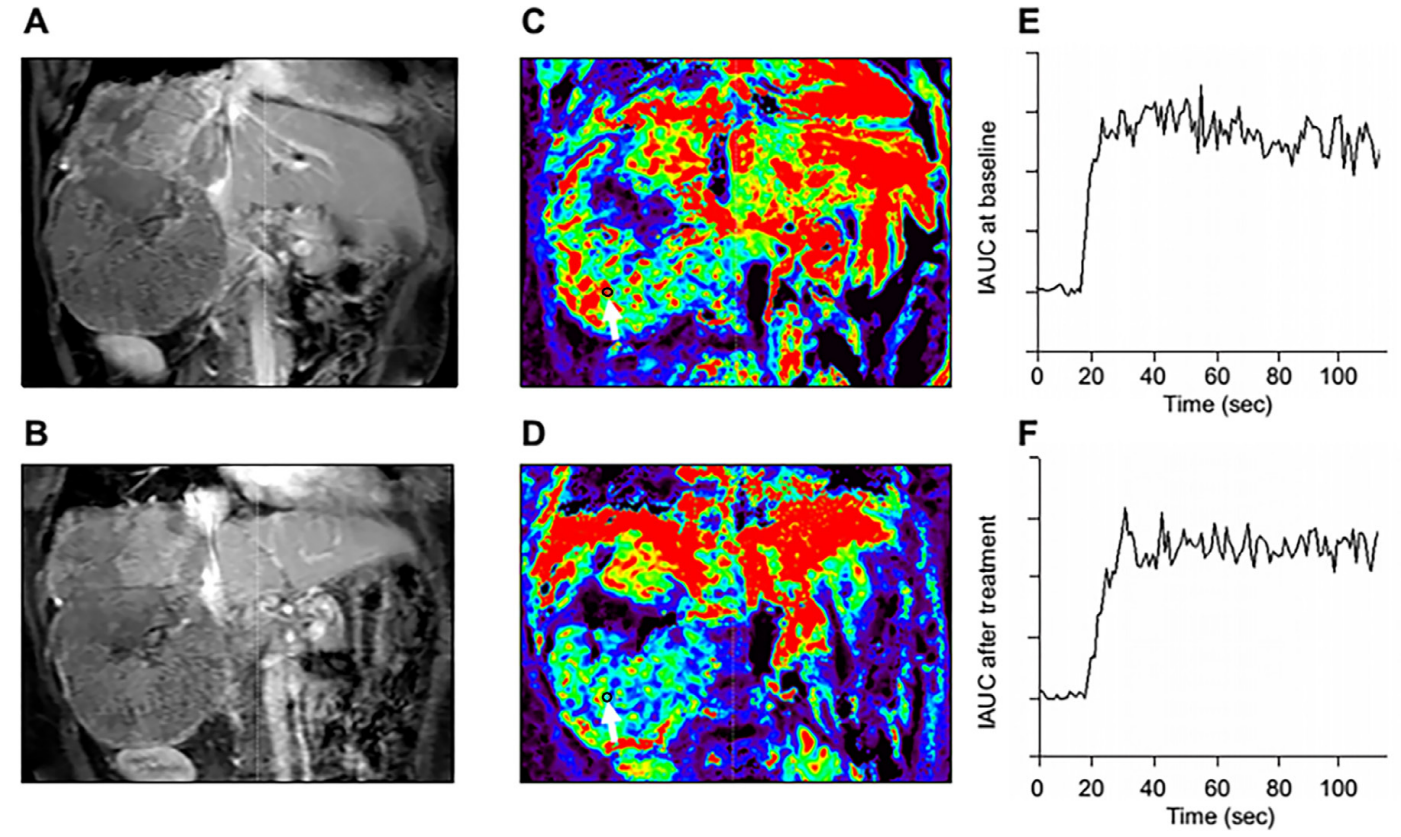
Representative DCE-MRI data in one advanced HCC patient **A**. Post-contrast T1-weighted MRI at baseline and **B**. after 14 days of study treatment. Color K^trans^ map of the tumors was greatly heterogeneous due to tumor necrosis and ROI was selected in the most enhanced tumor region. **C**. Corresponding color K^trans^ maps at baseline and **D**. after 14 days of study treatment. Hypervascular area is shown in red. The selected ROI (black cycle ) for K^trans^ measurement is indicated by white arrows, In this patient, the K^trans^ values at baseline and after study treatment were 798.6×10-3/min and 206.6 × 10-3/min, respectively. **E**. The initial area under the gadolinium concentration-time curves (IAUC) at baseline **F**. and after study treatment from the same patient. The IAUC values at baseline and after study treatment were 1526.2 mmol/kg×s and 1376.1 mmol/kg×s, respectively.

The ensemble of morpho-physiological characteristics of the tumour vessels provides the precise picture for predicting cancer outcomes [43]. Tumour cells pass through immature vessels into the blood stream, where they can be transported to remote organs. DCE-MRI with Gd-DTPA can predict tumor aggressiveness, as kinetic parameters such as K^trans^ and K_ep_ can discriminate low-grade from intermediate- or high-grade tumors in prostatic cancer [18]. Moreover, DCE-MRI accurately represents micro-vessel architecture and can distinguish low-grade from intermediate- and high-Gleason grade prostate tumors [44]. In the diagnostic of breast cancer, it was reported that DCE-MRI results closely correlated with anti-CD105 and anti-Ki67 data, as revealed by Pearson analysis, thus demonstrating that DCE-MRI could potentially be used for diagnosing early tumors [26]. Apparent diffusion coefficient (ADC) is an important factor of DWI, ADC is becoming a very useful biomarker for assessing tumor, response to a treatment with MRI technology, as ADC values increase significantly after a successful treatment. DCE-MRI has also been used with DWI to estimate necrosis in hepatocellular carcinoma after transcatheter arterial embolization, and to predict survival and response after anti-angiogenic therapy [22] (Figure 5). Moreover, some reports show that DCE-MRI can predict tumor response to radiotherapy, because this method can reproduce the tumor microenvironment variables [45].

### DCE-MRI with other low molecular contrast agents

Even though DCE-MRI with Gd-DTPA is widely used in preclinical and clinical research, some controversy remains as to whether this contrast agent with DCE-MRI can accurately reproduce the morpho-physiological and functional characteristics of tumor vessels. A number of studies have shown that low molecular weight agents can quickly overflow from vessels to the EES in both normal vessels and neovascularization, which could lead to an overestimation of vascular permeability [46, 47]. This raised the concern whether this method can distinguish normal and pathological vascular permeability and accurately reproduce vessel characteristics. However, some researchers suggest that more care should be taken in using low molecular contrast agents in preclinical research and that new contrast agents should be synthesized, others insist that low molecular contrast agents are suitable for describing vessel characteristics, including permeability. Nonetheless, considerable research efforts have been invested into finding alternative contrast agents for DCE-MRI. Zeng et al compared Gd-DTPA and albumin-binding Gd-EOB-DTPA for assessing microvessel characteristics with DCE-MRI in a murine orthotropic pancreatic cancer model and found that the tumor rim could be distinguished from the tumor core by Gd-EOB-DTPA but not Gd-DTPA, which was consistent with immunohistology data showing that CD31 and VEGF expression in the tumor rim was significantly higher than in the core [17].

Another study assessed contrast agents of different molecular weight in DCE-MRI, including Gd-DOTA (0.5 kDa), P846 (3.5 kDa), and P792 (6.47 kDa), showed that P792 was superior in distinguishing tumor from muscle [48]. DCE-MRI with P846 was also used for monitoring therapy-induced microvascular changes in a pancreatic cancer model. The method could detect pharmokinetic changes in K^trans^, V_p_, V_e_ after treated with gemcitabine, sunitinib or radiotherapy alone or in combination [49]. In addition, DCE-MRI with P846 could predict early small therapeutic effects. DCE-MRI with Gadodiamide could assess early response to bevacizumab therapy in breast cancer xenografts either alone or given in combination with doxorubicin. Moreover, this contrast agent accurately detected vascular normalization after treatment, showing that it could become a valuable tool for monitoring anti-angiogenesis in breast cancer [50]. A study compared the medium-sized contrast agent gadomer to the low molecular contrast agent Gd-DTPA in skeletal muscle concluded that for optimal estimation of microvascular parameters, both model-based and model-free analysis should be adapted to the pharmacokinetic properties of the contrast agent in order to increase the sensitivity of the detection [51].

In addition to the microvascular parameters mentioned above, clinical investigations have suggested that DCE-MRI may also be a useful method for assessing tumor interstitial fluid pressure (IFP). Interestingly, while low molecular weight contrast agents appear to have better correlation to IFP than higher molecular weight agents [52]. A review by Preda et al suggests that macromolecular contrast agents are more appropriate to monitor anti-angiogenesis [53]. Moreover, it has been shown that P792 is a superior contrast agent to Gd-DTPA for measuring IFP in cervical carcinoma xenografts [54] and detecting neovascularization [48].

Although low molecular contrast agents are used widely, there are disadvantages to overcome, including the precision of low molecular contrast agents in DCE-MRI applications. Further, clinical approved gadolinium contrast agents, particularly using linear chelates, have a potential of nephrogenic systemic fibrosis, due to transmetallation and loss of Gd (III) ions from the chelate. Enhancing relaxivity r1 to reduce gadolinium exposure remains an important approach to this issue.

## DCE-MRI with macromolecular contrast agents

Macromolecular MR contrast agents measure over 20kDa in molecular weight and are classed into three types based on their chemical structure: (i) albumin-binding gadolinium chelates, which simulate albumin transport from vessel to extracellular ; (ii) high polymeric compounds and (iii) biodegradable macromolecular compounds. Many believe that macromolecular contrast agents can better assess vascular characteristics because the pharmacodynamics models used in DCE-MRI to calculate vascular permeability are based on the properties of macromolecules such as albumin, which is often used as a standard. Macromolecular contrast agents are mainly explored in preclinical research for diagnosis and monitoring of anti-angiogenesis, but they cannot be used in clinical studies due to their biotoxicity until now.

### Albumin-binding contrast agents

Albumin-binding contrast agents are the most classical macromolecular contrast agents and are vastly used in preclinical tumor diagnosis and therapy response monitoring. These agents take advantage of albumin's normal vessel penetration process to calculate vascular permeability. DCE-MRI with albumin-(Gd-DTPA) 45 can detect the early effects on tumor microvasculature of a potentially curative treatment in experimental soft-tissue sarcomas [55]. Moreover, DCE-MRI monitoring of sorafenib therapy effects on experimental prostate carcinomas with albumin-(Gd-DTPA) 35 showed significant correlations with anti-angiogenic, anti-proliferative and proapoptotic effects determined immunohistochemically [56]. A DCE-MRI study assessing anti-PDGFR therapy effects in a prostate cancer bone metastasis model with biotin-labeled albumin-Gd-DTPA showed a reduction in vascular permeability and provided insights into the role played by VEGF in anti-PDGFR therapy [57]. In breast tumor models, DCE-MRI with albumin-Gd-DTPA revealed a decreasing tumor vascular permeability surface area product after anti-angiogenic bevacizumab/paclitaxel combination therapy [58]. While compared Gd-DTPA and MS-325, an albumin-binding contrast agent, both contrast agents could evaluate the stromal content in DU-145 or BXPC-3 cancers, but MS-325 showed greater dose-effectiveness than Gd-DTPA [59].

### Small molecular contrast agents that bind serum albumin

Some classes of small molecular contrast agents can reversible bind to serum albumin and form a macromolecular contrast medium (MMCM) after injection into the body. For instance, Gadofosveset is a Gd-based contrast agent that can bind reversibly to albumin thereby prolonging its vascular presence and increasing its relativity (r1) by 5 to 10 fold, however, when Gadofosveset was tested for assessing endothelial permeability in atherosclerosis it could not detect significant differences between normal and tumour vessels and showed low enhancement [60]. In contrast, B-22956/1 (86kDa), another contrast agent that binds reversibly to albumin, was enhanced 10 fold in the tumor rim after anti-angiogenesis therapy when compared to Gd-DTPA-albumin, and was also detected in the tumor core. Conversely, Gd-DTPA-albumin had a poor performance in detecting changes in the tumor core after therapy due to low enhancement and experimental error. These results may be explained by the size of the contrast agents, as Gd-based albumin-binding agents stay in the intercellular space longer than traditional agents, resulting in a longer time-window for enhancement and therefore producing a more accurate picture of angiogenesis. However, Gd-based albumin-binding agents have some disadvantages such as low value of permeability, low sensitivity to vascular changes after anti-angiogenesis therapy and high experiment error [61]. Another agent that can reversibly bind to albumin, MP-2269 [53], has not yet been tested in DCE-MRI.

### High polymer contrast agents

Macromolecular polymer contrast agents have higher r1 value than low molecular contrast agents, they are thought to correctly conform to pharmacokinetic models and have attracted vast research interest. For instance, it was shown that PEG-G3-(Gd-DTPA) 6-(cRGD-DTPA) 2 can target integrin avβ3 *in vivo* and assess early antiangiogenic effects before volume changes [62]. Moreover, a new macro cyclic MRI contrast agent, poly ( [(Gd-DOTA)-DETA]-co-DTBP) or (GODP), can detect the anti-angiogenic effects of bumetanide in a cancer colon model, including a significant reduction in the Fp and PS parameters after treatment. Importantly, CD31 and VEGF reduced expression detected by immunecytohistology in the tumor tissue confirmed these MRI results [63]. A study compared DCE-MRI performance of Gd-DTPA (0.55KDa), Gadomer-17 (30KDa) and polylysine-Gd-DTPA (50KDa) in mouse fibrosarcoma models found that Gd-DTPA produces the highest permeability-surface area (PS) values, however, the difference between tumor and normal tissue was not significant. In contrast, relatively high mean PS values were obtained in tumors with Gadomer-17 and Polylysine-Gd-DTPA when compared to normal tissue [64]. (CMD)-A2-Gd-DOTA is a slow-clearance contrast agent that remains in vessels for over one hour. Brasch et al tested the feasibility of using this new agent with DCE-MRI for distinguishing benign from malignant tumors and for tumor grading. However, no significant correlations were found between the MRI-estimated endothelial transfer coefficient and plasma volumes with histological tumor grade, possibly because the signal-to-background ratio (SBR) was not controlled in this experiment [65].

### Biodegradable macromolecular MRI contrast agents

Macromolecular contrast agents show better results in preclinical studies than traditional agents, however, they cannot be used in clinical research due to their slow excretion rates and immunotoxicity. To solve these issues, biodegradable macromolecular contrast agents have been synthesized which not only have characteristics of macromolecular agents, including a strong enhancement in vascular areas or tumor region, but also facilitated excretion of Gd (III) ions chelate. Zheng-Rong Lu's team did a remarkable work comparing biodegradable macromolecular agents of different sizes with other types of contrast agents including low molecular agents and Gd-DTPA-albumin in two tumor models. Interestingly, the K^trans^ values in both tumor models decreased as the molecular weight of the contrast agents increased, and agents with higher degradability showed higher K^trans^ values [66]. Moreover, degradability only had a significant impact on high molecular weights agents. The authors conclude that biodegradable macromolecular agents with high molecular weight could provide a more accurate assessment on tumor vascularity and angiogenesis with DCE-MRI than low molecular weight contrast agents [67]. Indeed, DCE-MRI with GDCC-40 (40kDa) could accurately detect early tumor response to indocyanine green-enhanced photothermic therapy and relapse after treatment [68]. To address the toxicity problem of albumin binding Gd (III) (ions), Cyran et al designed Gd-macromolecular contrast agents with a polyethylene glycol (PEG) core which were safe to humans and met the physicochemical and pharmacologic requirements for quantitative MRI characterization of blood vessels. A preliminary study showed that DCE-MRI with PEG12, 000-Gen4-(Gd-DOTA) 16 can accurately monitor the early anti-angiogenic effects of bevacizumab in a human melanoma model in rats [69].

In conclusion, macromolecular contrast agents provide more accurate quantitative assessments of angiogenesis than traditional low contrast agents in DCE-MRI and are also superior in diagnosing and monitoring the effects of cancer treatment. However, they present important disadvantages that preclude their application in clinical research. First, macromolecular agents cannot rapidly be excreted from the body and therefore accumulate in important organs, resulting in long-term toxicity. Second, albumin-bound Gd (III) ions have potential immune-toxicity in the plasma. Third, some macromolecular agents are not sensitive enough to detect changes in angiogenesis due to their intrinsic low values in quantitative parameters and associated high experiment error. On the other hand, low molecular weight Gd-chelates with reversible binding to plasma proteins could represent a new class of MRI contrast agents suitable for DCE-MRI. However, currently only two protein binding Gd-based contrast agents are considered for MRI angiography: MS-325 and B22956/1. Although low molecular weight Gd-DTPA is used most widely, macromolecular contrast agents can provide more precise DCE-MRI quantification data. It is essential to concentrate efforts on improving the accuracy and reliability of DCE-MRI for angiogenesis-based cancer diagnosis and monitoring of treatment effectiveness. Macromolecular contrast agents of 20kDa or larger seem to more promising, but toxicity and sensitivity issues need to be addressed in the future.

## DCE-MRI with hepatocyte-specific contrast agents

Hepatocyte-specific contrast agents partially enter hepatic parenchymal cells after intravenous injection and then are excreted from the biliary system while the rest remains in circulating blood. Gadoxetate (Gd-EOB-DTPA) is currently the most commonly used hepatocyte-specific agent. After bolus injection into vein, about 50% of this agent is taken up by functioning hepatocytes and then excreted through the biliary system, while the other 50% will return into blood vessels from the EES to be eliminated *via* kidney. Gd-EOB-DTPA therefore combines the features of an extracellular contrast agent and a hepatocyte-specific agent, to the great advantage that it can simultaneously provide morphological information of the liver and functional information of hepatocytes. This contrast agent has been extensively used in liver disease clinical research, particularly for hepatic carcinoma diagnosis and evaluation of liver function. For instance, Shih et al used DCE-MRI with Gd-EOB-DTPA to assess liver fibrosis using dual-input single-compartment and curve analysis models. After injection, most of the contrast agent remained in the intravascular and EES, from 60 s to 100 s post-injection it entered the hepatocytes and then a large amount accumulated in hepatocytes. Additionally, some Gd-EOB-DTPA left the liver *via* the hepatic vein and was excreted by the kidneys. The authors concluded that DCE-MRI with Gd-EOB-DTPA using multiple perfusion parameters was a suitable method for evaluating the severity of liver fibrosis [70]. Another study used DCE-MRI with Gd-EOB-DTPA to assess liver fibrosis found that K^trans^ and initial areas under the tissue concentration curves(iAUC) values obtained using this approach were accurate in detecting rat liver fibrosis induced by carbon tetrachloride [16]. Except Gd-EOB-DTPA, there are another hepatocyte-specific agent, like Gadobenate dimeglumine (Gd-BOPTA), superparamagnetic iron oxide (SPIO), Mn-DPDP. Despite these promising results, hepatocyte-specific contrast agents have limited clinical applications because they can enter hepatic parenchymal cells and traditional pharmacokinetics models are therefore not suitable. So there is needing an extraordinary pharmacokinetic model instead of traditional model. Nevertheless, hepatocyte-specific contrast agents are extremely useful for hepatic disease diagnosis [71–73] and monitoring of hepatic carcinoma [22, 74, 75].

## DCE-MRI with nanoparticle contrast agents

Nanoparticle research is an area of intense scientific research due to its many potential applications in various fields including biomedicine. The unique physical and chemical properties of materials at the nanometer scale allow the design of superior imaging probes with improved properties, for instance, contrast enhancement, increased sensitivity, better spatial and temporal information, and controlled biodistribution [76]. Currently, two types of nanoparticles can be used as MRI contrast agents: T1 contrast agents are nanoparticles with Gd (III), IN (II) Mn (II), and T2 contrast agents are nanoparticles with Fe3O4. T1 contrast agents such as tri-modal calcium phosphate nano-contrast agent, bound with indocyanine green (ICG) and Gd (III), and labeled with 99m-Technetium-methylene bisphosphonate (99mTc-MDP), showed potential for detecting liver angiogenesis in mouse models with excellent hemocompatibility and without any major histological changes in vital organ, and a clearance of 48 hours [77]. Moreover, biodegradable polysulfide dendrimer nanoclusters with a circulation half-life of > 1.6h in mice models produced significant contrast enhancement in the abdominal aorta and kidneys for as long as 4h, and then were degraded and eliminated *via* kidney without generation of free Gd(III) ions and without renal toxicity [78]. A new type of Gd (III) nano-contrast agent with an r1 value of 12.25mM-1S-1 and average size of 25 nm showed excellent MRI contrast properties and biocompatibility [79].

**Figure 6:**
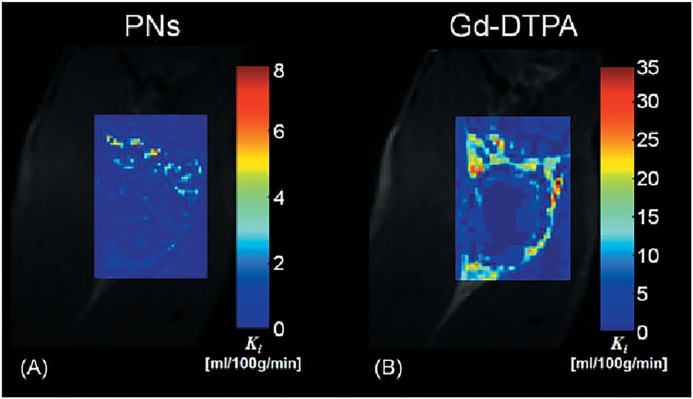
The trans endothelial transfer coefficient (Ki) maps produced from the PNs A. and Gd-DTPA B. The Ki map produced from PNs include discrete areas of high Ki within the tumor rim that are difficult to discern in the equivalent Gd-DTPA Ki map This is largely due to the small molecular size and considerably greater extraction of Gd-DTPA relative to PNs.

Research efforts have also been made to explore nanoparticles of T2 contrast agents. Weller et al synthesized and tested several different sizes of PEGylated iron-oxide-based negative MRI contrast agents [80]. Remarkably, Hosseini et al developed novel hybrid nanostructured systems that can both be used in medical imaging and drug delivery. Experiments *in vitro* showed that these new nanoparticles made of superparamagnetic iron oxide nanoparticles and pseudopolyrotaxanes behaved better in MRI than the ferumoxides [81]. In addition, there have been attempts to synthesize contrast agents with T1 and T2 dual mode MRI contrast enhanced function by combining different paramagnetic and super-paramagnetic materials [82]. However, while nanoparticles show great potential for clinical applications, few studies have assessed the feasibility of using DCE-MRI with nanoparticles to noninvasively evaluate angiogenesis. A study comparing the performance of Gd-DTPA and paramagnetic nanoparticles (225nm, lipid encapsulated, r1 1690000/s mM particle) for assessing angiogenesis with DCE-MRI found that the nanoparticles could differentiate areas of different angiogenesis in a rabbit Vx-2 tumor model [83] (Figure 6).

Nanoparticle-based contrast agents offer numerous advantages when compared to other types of agents, higher r1, lower dose Gd (III) needed, multifunctional. But many challenges remain to optimize these probes for clinical applications. First, nanoparticles are foreign bodies and as such can be eliminated by mononuclear phagocytes thereby increasing the critical amount of probe necessary for imaging. Second, nanoparticles have a complex composition and therefore may have high retention, which could result in toxicity. Third, all contrast agents need to have low toxicity for FDA approval. Very few new contrast agents are approved, particularly for nanoparticle contrast agents, because the cost of development and manufacture exceeds the reimbursement potential [76]. There is little research involving nanoparticle based DCE-MRI. To a great extent, paramagnetic nanoparticles are less effective as blood pool agents than small molecules, their advantage being targeted imaging where concentration of particles is able to overcome partial volume dilution.

## FUTURE DIRECTIONS

Research advances over the past decades show that angiogenesis can be assessed noninvasively using DCE-MRI. However, this method is widely used in tumor diagnosis and monitoring of treatment, choosing the proper contrast agent is essential to achieve accurate results. A proper MR contrast agent for DCE-MRI, first, it should have low toxicity, high r1, and low does to acquire optimal image, second, it should more precise with proper mathematical model to evaluate angiogenesis, third, it is easy to product with low cost of development and manufacture. There is currently a large variety of contrast agents available but no consensus as to which is best suited for DCE-MRI. Although macromolecular agents are thought to combine the optimal properties for accurate imaging, their potential toxicity compromises their use in clinical research. Recently, an increasing number of studies using nanoparticle-based contrast agents revealed their superior properties for DCE-MRI, including low toxicity and possibility for multimodal imaging. We hope that future research on nanoparticle-based contrast agents for DCE-MRI will produce ground-breaking developments.

## Abbreviations

DCE-MRI: Dynamic contrast-enhanced magnetic resonance imaging
VEGF: vascular endothelial growth factor
EES: extravascular extracellular space
ROI: region-of-interest
SI: signal intensity
ADC: apparent diffusion coefficient
MTT: mean transit time
Gd-DTPA: gadopentetate dimeglumine
DWI: diffusion weight imaging
TTP: time to peak
3D-CEUS: three-dimensional contrast enhanced ultrasound
IFP: interstitial fluid pressure
MMCM: macromolecular contrast medium
SBR: signal-to-background ratio
iAUC: initial areas under the tissue concentration curves
SPIO: superparamagnetic iron oxide.
